# 2-Methyl-3-(5-methyl-2-thien­yl)-5-phenyl­perhydro­pyrrolo[3,4-*d*]isoxazole-4,6-dione

**DOI:** 10.1107/S1600536809009209

**Published:** 2009-03-25

**Authors:** Mustafa Odabaşoğlu, Hamdi Özkan, Yılmaz Yıldırır, Orhan Büyükgüngör

**Affiliations:** a Pamukkale University, Denizli Higher Vocational School, Chemistry Program, TR-20159 Kınıklı, Denizli, Turkey; b Department of Chemistry, Faculty of Arts and Science, Kırıkkale University, Kırıkkale, Turkey; c Department of Chemistry, Faculty of Arts and Science, Gazi University, Ankara, Turkey; d Department of Physics, Faculty of Arts and Science, Ondokuz Mayıs University, TR-55139 Kurupelit Samsun, Turkey

## Abstract

In the mol­ecule of the title compound, C_17_H_16_N_2_O_3_S, the phenyl ring is oriented with respect to the thio­phene and succinimide rings at dihedral angles of 88.08 (3) and 57.81 (3)°, respectively; the dihedral angle between the thio­phene and succinimide rings is 35.69 (3)°. The isoxazole ring adopts an envelope conformation with the N atom at the flap position. In the crystal structure, inter­molecular C—H⋯O hydrogen bonds link the mol­ecules into infinite chains along the *b* axis. Weak C—H⋯π inter­actions may further stabilize the structure.

## Related literature

For nitrones as versatile synthetic intermediates in organic synthesis, see: Black *et al.* (1975 ▶); Banerji & Sahu (1986 ▶); Torsell (1988 ▶); Banerji & Basu (1992 ▶). For nitrones as a convenient class of compounds for the syntheses of ultimate carcinogens, see: Mallesha, Ravikumar, Mantelingu *et al.* (2001 ▶); Mallesha, Ravikumar & Rangappa (2001 ▶). For the 1,3-dipolar cycloaddition reaction of nitrones with alkenes in the preparation of isoxazolidines, see: Tufariello (1984 ▶). For isoxazolidines in the synthesis of β-lactams, see: Padwa *et al.* (1981 ▶, 1984 ▶). For the use of β-lactams to treat bacterial infections, see: Ochiai *et al.* (1967 ▶); as natural products, see: Baldwin & Aube (1987 ▶); as versatile synthetic intermediates, see: Padwa (1984 ▶). For the preparation of *C*-(5-Methyl-2-thienyl)-*N*-methylnitrone used in the synthesis, see: Heaney *et al.* (2001 ▶). For bond-length data, see: Allen *et al.* (1987 ▶).
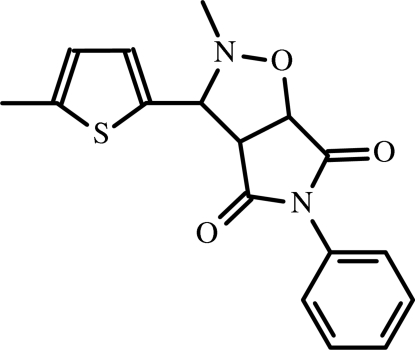


## Experimental

### 

#### Crystal data

C_17_H_16_N_2_O_3_S
*M* *_r_* = 328.38
Monoclinic, 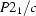

*a* = 12.6558 (5) Å
*b* = 8.5738 (3) Å
*c* = 19.3824 (8) Å
β = 128.654 (3)°
*V* = 1642.42 (13) Å^3^
*Z* = 4
Mo *K*α radiation
μ = 0.21 mm^−1^
*T* = 296 K
0.73 × 0.52 × 0.26 mm

#### Data collection

STOE IPDS 2 diffractometer
Absorption correction: integration (*X-RED32*; Stoe & Cie, 2002 ▶) *T*_min_ = 0.685, *T*_max_ = 0.946
19464 measured reflections
3401 independent reflections
2872 reflections with *I* > 2σ(*I*)
*R*_int_ = 0.028

#### Refinement

*R*[*F* ^2^ > 2σ(*F* ^2^)] = 0.037
*wR*(*F* ^2^) = 0.098
*S* = 1.05
3401 reflections
222 parameters
H atoms treated by a mixture of independent and constrained refinement
Δρ_max_ = 0.19 e Å^−3^
Δρ_min_ = −0.27 e Å^−3^

### 

Data collection: *X-AREA* (Stoe & Cie, 2002 ▶); cell refinement: *X-AREA*; data reduction: *X-RED32* (Stoe & Cie, 2002 ▶); program(s) used to solve structure: *SHELXS97* (Sheldrick, 2008 ▶); program(s) used to refine structure: *SHELXL97* (Sheldrick, 2008 ▶); molecular graphics: *ORTEP-3 for Windows* (Farrugia, 1997 ▶); software used to prepare material for publication: *WinGX* (Farrugia, 1999 ▶).

## Supplementary Material

Crystal structure: contains datablocks I, global. DOI: 10.1107/S1600536809009209/hk2634sup1.cif

Structure factors: contains datablocks I. DOI: 10.1107/S1600536809009209/hk2634Isup2.hkl

Additional supplementary materials:  crystallographic information; 3D view; checkCIF report

## Figures and Tables

**Table 1:** Hydrogen-bond geometry (Å, °)

*D*—H⋯*A*	*D*—H	H⋯*A*	*D*⋯*A*	*D*—H⋯*A*
C8—H18⋯O2^i^	0.94 (2)	2.41 (2)	3.103 (2)	130
C2—H2⋯*Cg*1^ii^	0.93	2.99	3.83 (2)	152 (1)

Symmetry codes: (i) 
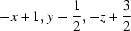
; (ii) 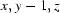. *Cg*1 is the centroid of the S1/C13–C16 ring.

**Fig. 1.**
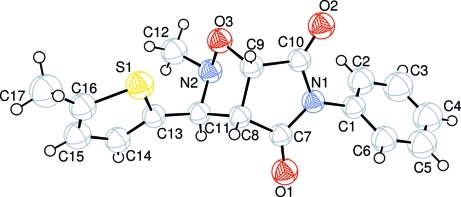
The molecular structure of the title molecule, with the atom-numbering scheme.

**Fig. 2.**
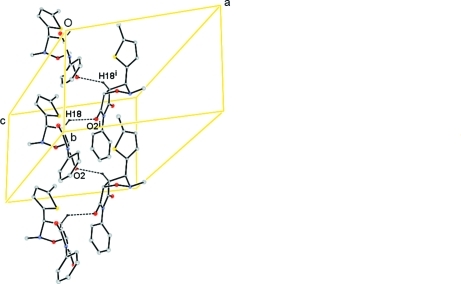
A partial packing diagram of the title compound [symmetry code: (i) 1-x, y+1/2, z]. Hydrogen bonds are shown as dashed lines. H atoms not involved in hydrogen bonding have been omitted.

**Fig. 3.**
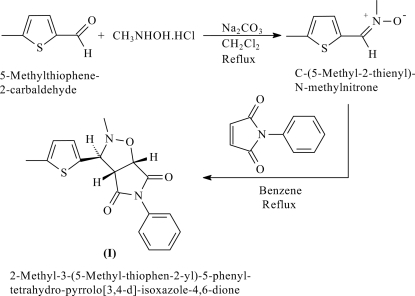
The formation of the title compound.

## supplementary crystallographic information

### Comment

1,3-Dipolar cycloaddition reaction of nitrones to olefins is of synthetic interest. In the present work, isoxazolidines have been synthesized in high yield *via* intermolecular cycloaddition of *N*-arylnitrone with monosubstituted olefins and are employed for biological evaluation. Nitrones are versatile synthetic intermediates in organic synthesis (Black *et al.*, 1975; Banerji & Sahu, 1986; Torsell, 1988; Banerji & Basu, 1992). Recently, they reported that nitrones are a convenient class of compounds for the syntheses of ultimate carcinogens (Mallesha, Ravikumar, Mantelingu *et al.*, 2001); Mallesha, Ravikumar & Rangappa, 2001), which are biologically interesting molecules. The 1,3-dipolar cycloaddition reaction of nitrones with alkenes is an important method for preparing isoxazolidines in a regioselective and stereoselective manner (Tufariello, 1984). These isoxazolidines are used in the syntheses of β-lactams (Padwa *et al.*, 1981; Padwa *et al.*, 1984) which are of value in the treatment of bacterial infections (Ochiai *et al.*, 1967), occur as natural products (Baldwin & Aube, 1987), serve as versatile synthetic intermediates (Padwa, 1984), and are biologically interesting compounds (Ochiai *et al.*, 1967). In view of the interest shown in these compounds, we report herein the crystal structure of the title compound.

In the molecule of the title compound (Fig. 1), the bond lengths (Allen *et al.*,  1987) and angles are within normal ranges. Rings A (C1-C6), B (N1/C7-C10) and D (S1/C13-C16) are, of course, planar, and they are oriented at dihedral angles of A/B = 57.81 (3), A/D = 88.08 (3) and B/D = 35.69 (3) °. Ring C (O3/N2/C8/C9/C11) adopts envelope conformation with N2 atom displaced by 0.736 (3) Å from the plane of the other ring atoms.

In the crystal structure, intermolecular C-H···O hydrogen bonds (Table 1) link the molecules (Fig. 2) into infinite chains along the b-axis, in which they may be effective in the stabilization of the structure. The weak C—H···π interaction (Table 1) may further stabilize the structure.

### Experimental

C-(5-Methyl-2-thienyl)-N-methylnitrone was prepared from 5-methylthiophene-2 -carbaldehyde, N-methyl-hydroxylamine hydrochloride and sodium carbonate in CH_2_Cl_2_ (Scheme 2) according to the literature method (Heaney *et al.*, 2001). For the preparation of the title compound, C-(5-methyl-2-thienyl)-N -methylnitrone (471 mg, 3 mmol) and N-phenylmaleimide (570 mg, 3.3 mmol) were dissolved in benzene (50 ml). The reaction mixture was refluxed for 12 h, and monitored by TLC (Scheme 2). After evaporation of the solvent, the reaction mixture was separated by column chromatography, using mixtures of petroleum ether and ethyl acetate (1:2) as the eluant. The *trans*-isomer, was recrystallized from CHCl_3_/n-hexane (1:3) in 2 d (m.p. 425-428 K).

### Refinement

Atoms H18, H19 and H20 were located in difference synthesis and refined isotropically [C-H = 0.941 (16)-0.974 (17) Å and U_iso_(H) = 0.049 (4)-0.061 (5) Å^2^]. Remaining H atoms were positioned geometrically, with C-H = 0.93 and 0.96 Å for aromatic and methyl H, respectively, and constrained to ride on their parent atoms, with U_iso_(H) = xU_eq_(C), where x = 1.5 for methyl H and x = 1.2 for aromatic H atoms.

### Crystal data

**Table:**

C_17_H_16_N_2_O_3_S	*F*(000) = 688
*M**_r_* = 328.38	*D*_x_ = 1.328 Mg m^−^^3^
Monoclinic, *P*2_1_/*c*	Mo *K*α radiation, λ = 0.71073 Å
Hall symbol: -P 2ybc	Cell parameters from 19464 reflections
*a* = 12.6558 (5) Å	θ = 1.6–28.0°
*b* = 8.5738 (3) Å	µ = 0.21 mm^−^^1^
*c* = 19.3824 (8) Å	*T* = 296 K
β = 128.654 (3)°	Prism, colorless
*V* = 1642.42 (13) Å^3^	0.73 × 0.52 × 0.26 mm
*Z* = 4	

### Data collection

**Table:**

STOE IPDS 2 diffractometer	3401 independent reflections
Radiation source: sealed X-ray tube, 12 x 0.4 mm long-fine focus	2872 reflections with *I* > 2σ(*I*)
plane graphite	*R*_int_ = 0.028
Detector resolution: 6.67 pixels mm^-1^	θ_max_ = 26.5°, θ_min_ = 2.1°
ω–scan rotation method	*h* = −15→15
Absorption correction: integration (*X-RED32*; Stoe & Cie, 2002)	*k* = −10→10
*T*_min_ = 0.685, *T*_max_ = 0.946	*l* = −24→24
19464 measured reflections	

### Refinement

**Table:**

Refinement on *F*^2^	Primary atom site location: structure-invariant direct methods
Least-squares matrix: full	Secondary atom site location: difference Fourier map
*R*[*F*^2^ > 2σ(*F*^2^)] = 0.037	Hydrogen site location: inferred from neighbouring sites
*wR*(*F*^2^) = 0.098	H atoms treated by a mixture of independent and constrained refinement
*S* = 1.05	*w* = 1/[σ^2^(*F*_o_^2^) + (0.0494*P*)^2^ + 0.2689*P*] where *P* = (*F*_o_^2^ + 2*F*_c_^2^)/3
3401 reflections	(Δ/σ)_max_ = 0.001
222 parameters	Δρ_max_ = 0.19 e Å^−^^3^
0 restraints	Δρ_min_ = −0.27 e Å^−^^3^

### Special details

**Table:**

Geometry. All e.s.d.'s (except the e.s.d. in the dihedral angle between two l.s. planes) are estimated using the full covariance matrix. The cell e.s.d.'s are taken into account individually in the estimation of e.s.d.'s in distances, angles and torsion angles; correlations between e.s.d.'s in cell parameters are only used when they are defined by crystal symmetry. An approximate (isotropic) treatment of cell e.s.d.'s is used for estimating e.s.d.'s involving l.s. planes.
Refinement. Refinement of *F*^2^ against ALL reflections. The weighted *R*-factor *wR* and goodness of fit *S* are based on *F*^2^, conventional *R*-factors *R* are based on *F*, with *F* set to zero for negative *F*^2^. The threshold expression of *F*^2^ > σ(*F*^2^) is used only for calculating *R*-factors(gt) *etc*. and is not relevant to the choice of reflections for refinement. *R*-factors based on *F*^2^ are statistically about twice as large as those based on *F*, and *R*- factors based on ALL data will be even larger.

### Fractional atomic coordinates and isotropic or equivalent isotropic displacement parameters (Å^2^)

**Table:**

	*x*	*y*	*z*	*U*_iso_*/*U*_eq_	
S1	0.31342 (4)	0.24446 (5)	0.79541 (3)	0.06492 (15)	
O1	0.18469 (12)	0.56188 (13)	0.50732 (7)	0.0639 (3)	
O2	0.42647 (11)	0.86900 (14)	0.75274 (7)	0.0629 (3)	
O3	0.28425 (11)	0.60569 (12)	0.76942 (7)	0.0549 (3)	
N1	0.30675 (12)	0.74300 (13)	0.61858 (8)	0.0479 (3)	
N2	0.14465 (13)	0.58932 (14)	0.68562 (9)	0.0527 (3)	
C1	0.29019 (15)	0.87293 (17)	0.56598 (9)	0.0509 (3)	
C2	0.2287 (2)	1.0061 (2)	0.56436 (13)	0.0712 (5)	
H2	0.1985	1.0126	0.5974	0.085*	
C3	0.2120 (3)	1.1305 (2)	0.51308 (15)	0.0896 (7)	
H3	0.1707	1.2213	0.5119	0.108*	
C4	0.2556 (3)	1.1214 (3)	0.46425 (15)	0.0889 (6)	
H4	0.2436	1.2055	0.4297	0.107*	
C5	0.3173 (2)	0.9880 (3)	0.46614 (13)	0.0814 (6)	
H5	0.3473	0.9821	0.4330	0.098*	
C6	0.33513 (17)	0.8621 (2)	0.51722 (11)	0.0628 (4)	
H6	0.3768	0.7715	0.5186	0.075*	
C7	0.25037 (14)	0.59615 (17)	0.58428 (9)	0.0473 (3)	
C8	0.28380 (14)	0.49173 (17)	0.65825 (9)	0.0460 (3)	
H18	0.3330 (16)	0.4050 (19)	0.6624 (10)	0.053 (4)*	
C9	0.36272 (14)	0.59533 (18)	0.74030 (9)	0.0490 (3)	
H20	0.4521 (18)	0.5567 (19)	0.7893 (11)	0.061 (5)*	
C10	0.37259 (13)	0.75292 (18)	0.70902 (9)	0.0487 (3)	
C11	0.15792 (14)	0.44699 (17)	0.64828 (9)	0.0472 (3)	
H19	0.0810 (16)	0.4439 (17)	0.5872 (10)	0.049 (4)*	
C12	0.05862 (19)	0.5727 (2)	0.71120 (13)	0.0675 (5)	
H12A	0.0615	0.6671	0.7390	0.101*	
H12B	0.0905	0.4873	0.7518	0.101*	
H12C	−0.0328	0.5527	0.6596	0.101*	
C13	0.17079 (14)	0.29515 (17)	0.69109 (9)	0.0490 (3)	
C14	0.07838 (16)	0.17956 (19)	0.65756 (11)	0.0585 (4)	
H14	−0.0060	0.1843	0.6020	0.070*	
C15	0.12214 (19)	0.0503 (2)	0.71510 (13)	0.0654 (4)	
H15	0.0695	−0.0383	0.7005	0.079*	
C16	0.2461 (2)	0.06732 (18)	0.79239 (11)	0.0611 (4)	
C17	0.3246 (3)	−0.0399 (2)	0.87043 (13)	0.0863 (6)	
H17A	0.3342	0.0069	0.9191	0.104*	
H17B	0.4125	−0.0580	0.8868	0.104*	
H17C	0.2774	−0.1372	0.8554	0.104*	

### Atomic displacement parameters (Å^2^)

**Table:**

	*U*^11^	*U*^22^	*U*^33^	*U*^12^	*U*^13^	*U*^23^
S1	0.0661 (3)	0.0518 (2)	0.0513 (2)	−0.00077 (18)	0.0242 (2)	0.00449 (17)
O1	0.0760 (7)	0.0603 (7)	0.0466 (6)	−0.0122 (5)	0.0340 (6)	−0.0086 (5)
O2	0.0621 (6)	0.0689 (7)	0.0590 (6)	−0.0249 (5)	0.0385 (5)	−0.0200 (5)
O3	0.0620 (6)	0.0565 (6)	0.0547 (6)	−0.0088 (5)	0.0406 (5)	−0.0077 (5)
N1	0.0488 (6)	0.0494 (6)	0.0469 (6)	−0.0058 (5)	0.0306 (5)	−0.0050 (5)
N2	0.0511 (7)	0.0495 (7)	0.0634 (7)	0.0039 (5)	0.0386 (6)	0.0026 (6)
C1	0.0514 (8)	0.0511 (8)	0.0498 (8)	−0.0087 (6)	0.0314 (7)	−0.0057 (6)
C2	0.0973 (13)	0.0549 (9)	0.0763 (11)	0.0028 (9)	0.0615 (11)	−0.0019 (8)
C3	0.1285 (19)	0.0525 (10)	0.0891 (14)	0.0070 (11)	0.0685 (14)	0.0019 (10)
C4	0.1185 (18)	0.0681 (12)	0.0788 (13)	−0.0123 (12)	0.0610 (13)	0.0090 (10)
C5	0.0878 (13)	0.0980 (15)	0.0694 (11)	−0.0068 (12)	0.0544 (11)	0.0105 (11)
C6	0.0620 (9)	0.0733 (11)	0.0584 (9)	0.0005 (8)	0.0401 (8)	0.0018 (8)
C7	0.0444 (7)	0.0486 (7)	0.0483 (7)	−0.0017 (6)	0.0286 (6)	−0.0046 (6)
C8	0.0421 (7)	0.0459 (7)	0.0460 (7)	0.0033 (6)	0.0257 (6)	−0.0006 (6)
C9	0.0412 (7)	0.0571 (8)	0.0433 (7)	0.0011 (6)	0.0237 (6)	−0.0017 (6)
C10	0.0391 (6)	0.0587 (8)	0.0482 (7)	−0.0080 (6)	0.0273 (6)	−0.0084 (6)
C11	0.0422 (7)	0.0481 (7)	0.0449 (7)	0.0011 (6)	0.0242 (6)	0.0014 (6)
C12	0.0724 (11)	0.0608 (10)	0.0930 (13)	0.0054 (8)	0.0633 (11)	0.0042 (9)
C13	0.0488 (7)	0.0464 (7)	0.0502 (8)	0.0001 (6)	0.0301 (6)	−0.0006 (6)
C14	0.0538 (8)	0.0563 (9)	0.0629 (9)	−0.0062 (7)	0.0352 (7)	−0.0017 (7)
C15	0.0780 (11)	0.0516 (9)	0.0828 (12)	−0.0090 (8)	0.0581 (10)	−0.0012 (8)
C16	0.0862 (12)	0.0480 (8)	0.0642 (10)	0.0071 (8)	0.0542 (10)	0.0048 (7)
C17	0.1304 (19)	0.0610 (11)	0.0754 (12)	0.0163 (11)	0.0682 (13)	0.0158 (9)

### Geometric parameters (Å, °)

**Table:**

C1—C2	1.371 (2)	C10—O2	1.2049 (18)
C1—C6	1.377 (2)	C10—N1	1.3963 (18)
C1—N1	1.4345 (18)	C11—N2	1.4809 (19)
C2—C3	1.381 (3)	C11—C13	1.497 (2)
C2—H2	0.9300	C11—H19	0.954 (15)
C3—C4	1.363 (3)	C12—N2	1.458 (2)
C3—H3	0.9300	C12—H12A	0.9600
C4—C5	1.372 (3)	C12—H12B	0.9600
C4—H4	0.9300	C12—H12C	0.9600
C5—C6	1.385 (3)	C13—C14	1.350 (2)
C5—H5	0.9300	C13—S1	1.7252 (15)
C6—H6	0.9300	C14—C15	1.417 (2)
C7—O1	1.2056 (17)	C14—H14	0.9300
C7—N1	1.3942 (18)	C15—C16	1.338 (3)
C7—C8	1.509 (2)	C15—H15	0.9300
C8—C9	1.5269 (19)	C16—C17	1.497 (2)
C8—C11	1.529 (2)	C16—S1	1.7243 (17)
C8—H18	0.941 (16)	C17—H17A	0.9600
C9—O3	1.4188 (18)	C17—H17B	0.9600
C9—C10	1.518 (2)	C17—H17C	0.9600
C9—H20	0.974 (17)	N2—O3	1.4813 (16)
			
C2—C1—C6	120.81 (16)	N2—C11—C8	99.14 (11)
C2—C1—N1	119.46 (14)	C13—C11—C8	113.83 (12)
C6—C1—N1	119.72 (14)	N2—C11—H19	106.8 (9)
C1—C2—C3	119.26 (18)	C13—C11—H19	109.7 (9)
C1—C2—H2	120.4	C8—C11—H19	109.8 (9)
C3—C2—H2	120.4	N2—C12—H12A	109.5
C4—C3—C2	120.6 (2)	N2—C12—H12B	109.5
C4—C3—H3	119.7	H12A—C12—H12B	109.5
C2—C3—H3	119.7	N2—C12—H12C	109.5
C3—C4—C5	119.98 (19)	H12A—C12—H12C	109.5
C3—C4—H4	120.0	H12B—C12—H12C	109.5
C5—C4—H4	120.0	C14—C13—C11	127.58 (14)
C4—C5—C6	120.29 (19)	C14—C13—S1	109.68 (12)
C4—C5—H5	119.9	C11—C13—S1	122.73 (11)
C6—C5—H5	119.9	C13—C14—C15	113.59 (15)
C1—C6—C5	119.05 (17)	C13—C14—H14	123.2
C1—C6—H6	120.5	C15—C14—H14	123.2
C5—C6—H6	120.5	C16—C15—C14	113.85 (15)
O1—C7—N1	124.20 (14)	C16—C15—H15	123.1
O1—C7—C8	126.75 (13)	C14—C15—H15	123.1
N1—C7—C8	109.05 (11)	C15—C16—C17	129.64 (18)
C7—C8—C9	104.90 (12)	C15—C16—S1	110.06 (12)
C7—C8—C11	112.15 (11)	C17—C16—S1	120.29 (16)
C9—C8—C11	103.27 (11)	C16—C17—H17A	109.5
C7—C8—H18	109.1 (10)	C16—C17—H17B	109.5
C9—C8—H18	113.8 (10)	H17A—C17—H17B	109.5
C11—C8—H18	113.2 (10)	C16—C17—H17C	109.5
O3—C9—C10	110.85 (12)	H17A—C17—H17C	109.5
O3—C9—C8	106.60 (11)	H17B—C17—H17C	109.5
C10—C9—C8	105.39 (11)	C7—N1—C10	112.34 (12)
O3—C9—H20	108.0 (10)	C7—N1—C1	123.94 (12)
C10—C9—H20	110.9 (10)	C10—N1—C1	123.56 (12)
C8—C9—H20	115.0 (10)	C12—N2—C11	114.98 (12)
O2—C10—N1	124.40 (14)	C12—N2—O3	105.60 (12)
O2—C10—C9	127.31 (13)	C11—N2—O3	101.08 (10)
N1—C10—C9	108.29 (12)	C9—O3—N2	102.11 (10)
N2—C11—C13	116.88 (12)	C16—S1—C13	92.81 (8)
			
C6—C1—C2—C3	0.0 (3)	S1—C13—C14—C15	−0.39 (18)
N1—C1—C2—C3	−179.52 (17)	C13—C14—C15—C16	0.8 (2)
C1—C2—C3—C4	0.2 (3)	C14—C15—C16—C17	177.85 (17)
C2—C3—C4—C5	−0.3 (4)	C14—C15—C16—S1	−0.8 (2)
C3—C4—C5—C6	0.3 (3)	O1—C7—N1—C10	177.30 (14)
C2—C1—C6—C5	0.0 (3)	C8—C7—N1—C10	−2.03 (16)
N1—C1—C6—C5	179.42 (15)	O1—C7—N1—C1	1.9 (2)
C4—C5—C6—C1	−0.1 (3)	C8—C7—N1—C1	−177.45 (12)
O1—C7—C8—C9	−177.41 (15)	O2—C10—N1—C7	−178.38 (14)
N1—C7—C8—C9	1.90 (15)	C9—C10—N1—C7	1.26 (15)
O1—C7—C8—C11	−66.0 (2)	O2—C10—N1—C1	−2.9 (2)
N1—C7—C8—C11	113.29 (13)	C9—C10—N1—C1	176.71 (12)
C7—C8—C9—O3	116.74 (12)	C2—C1—N1—C7	119.20 (17)
C11—C8—C9—O3	−0.88 (14)	C6—C1—N1—C7	−60.3 (2)
C7—C8—C9—C10	−1.12 (14)	C2—C1—N1—C10	−55.7 (2)
C11—C8—C9—C10	−118.74 (12)	C6—C1—N1—C10	124.80 (16)
O3—C9—C10—O2	64.68 (19)	C13—C11—N2—C12	−40.09 (18)
C8—C9—C10—O2	179.63 (14)	C8—C11—N2—C12	−162.79 (13)
O3—C9—C10—N1	−114.96 (12)	C13—C11—N2—O3	73.09 (14)
C8—C9—C10—N1	0.00 (15)	C8—C11—N2—O3	−49.61 (12)
C7—C8—C11—N2	−81.54 (13)	C10—C9—O3—N2	84.42 (12)
C9—C8—C11—N2	30.87 (13)	C8—C9—O3—N2	−29.78 (13)
C7—C8—C11—C13	153.59 (12)	C12—N2—O3—C9	170.81 (12)
C9—C8—C11—C13	−93.99 (14)	C11—N2—O3—C9	50.71 (12)
N2—C11—C13—C14	109.49 (18)	C15—C16—S1—C13	0.48 (14)
C8—C11—C13—C14	−135.77 (16)	C17—C16—S1—C13	−178.30 (15)
N2—C11—C13—S1	−70.16 (16)	C14—C13—S1—C16	−0.04 (13)
C8—C11—C13—S1	44.58 (17)	C11—C13—S1—C16	179.67 (13)
C11—C13—C14—C15	179.92 (15)		

### Hydrogen-bond geometry (Å, °)

**Table:**

*D*—H···*A*	*D*—H	H···*A*	*D*···*A*	*D*—H···*A*
C8—H18···O2^i^	0.94 (2)	2.41 (2)	3.103 (2)	130.0
C2—H2···Cg1^ii^	0.93	2.99	3.83 (2)	152 (1)

Symmetry codes: (i) −*x*+1, *y*−1/2, −*z*+3/2; (ii) *x*, *y*−1, *z*.
